# Bilateral Cervical Facet Dislocation Due to Catastrophic Shallow Water Diving: A Case Report

**DOI:** 10.7759/cureus.48846

**Published:** 2023-11-15

**Authors:** Yusoff Norisyam, Han Sim Lim, Zairul Bahrin, Choong Hoon Foo

**Affiliations:** 1 Spine Surgery, Hospital Pulau Pinang, George Town, MYS; 2 Orthopaedics, Hospital Queen Elizabeth, Kota Kinabalu, MYS

**Keywords:** diving injury, closed reduction, incomplete neurology, cervical spine, facet dislocation

## Abstract

Bilateral cervical facet dislocation is a rare injury resulting from headfirst shallow water diving accidents. Accurate diagnosis, prompt management, precise intervention, and aggressive rehabilitation can lead to a favourable neurologic and functional outcome for cervical spine injuries. In this case, we present a young adolescent patient who experienced bilateral facet dislocation of C4/C5, resulting in incomplete central cord syndrome neurological deficits (American Spinal Injury Association (ASIA) Impairment Scale C) due to a dangerous shallow water diving accident. The patient subsequently underwent emergency posterior instrumentation and decompression for stabilization and rehabilitation. Immediately following the surgery, he exhibited substantial neurologic recovery and was able to walk independently after six months. This case is unique not only for its rarity but also because it involved a young adolescent, highlighting the need for increased awareness and preventive measures to reduce the risk of dangerous shallow water diving accidents.

## Introduction

Diving into shallow water is the third most common mechanism of injury, contributing to 9.5% of cervical injuries, following motor vehicle accidents and falls from heights [1]. Facet dislocation injuries of the cervical spine are rare, accounting for less than 10% of all cervical spine injuries, and are strongly associated with neurological deficits, often resulting in significant and permanent disability [2]. Diving accidents represent an uncommon mechanism of facet dislocation and occur due to extreme flexion of the cervical spine with axial loading, and their rarity is reflected in the limited number of reported cases, less than 2% [1]. Given the severe neurological consequences, which can lead to permanent disability, and the substantial sociological and financial burden on society, it is crucial to focus on better prevention strategies to mitigate the impact of this public health problem.

## Case presentation

A 13-year-old boy with no comorbidities arrived at the emergency department following an injury sustained while diving headfirst into a shallow river, where he collided with a rock at a depth of 1.5 meters. Post trauma, he experienced neck pain, bilateral upper and lower limb weakness, and numbness. His friends rescued him from drowning due to his weakness, and he was unable to move his limbs. Fortunately, he displayed no scalp wounds, loss of consciousness, vomiting, or seizures, and there was no urinary or bladder incontinence.

Upon examination in the emergency department, he was fully conscious with a Glasgow Coma Scale (GCS) of 15, a normal body temperature, and stable vital signs. The neck examination revealed midline posterior tenderness, a step deformity in the mid-cervical spine, and limited range of motion due to pain. No abnormalities were found in the chest or pelvic areas, and there were no observed long bone deformities.

The initial neurological examination showed that the patient was in spinal shock, with complete neurological deficits in both upper and lower limbs. Muscle power, as measured by the Medical Research Council (MRC) scale, was rated at 0, and the sensory level was C5, with no sacral sparing and an absence of the bulbocavernosus reflex.

A subsequent neurological examination 24 hours later showed the resolution of spinal shock and the return of the bulbocavernosus reflex. The new neurological examination indicated an incomplete cervical cord injury with central cord syndrome (American Spinal Injury Association (ASIA) Impairment Scale C). Muscle power for bilateral C5 was graded 5 on the MRC scale, with bilateral C6 rated at 0, bilateral C7 at 1, and bilateral C8 and T1 at 0. Muscle power for the lower limbs was stronger compared to the upper limbs, with bilateral L2 rated at MRC grade 1, bilateral L3 at 2, right L4 at 4 compared to left L4 at 1, right L5 at 4 compared to left L4 at 3, and right S1 at 5 compared to left S1 at 3. Sensation was reduced from C4 downward, and hypertonia and hyperreflexia were observed in both upper and lower limbs. A per-rectal examination revealed intact voluntary anal contraction and the presence of deep anal pressure sensation. Other systemic examinations showed no abnormalities. Baseline blood parameters revealed normal findings for both full blood count and renal profiles.

Diagnostic assessment

**Figure 1:**
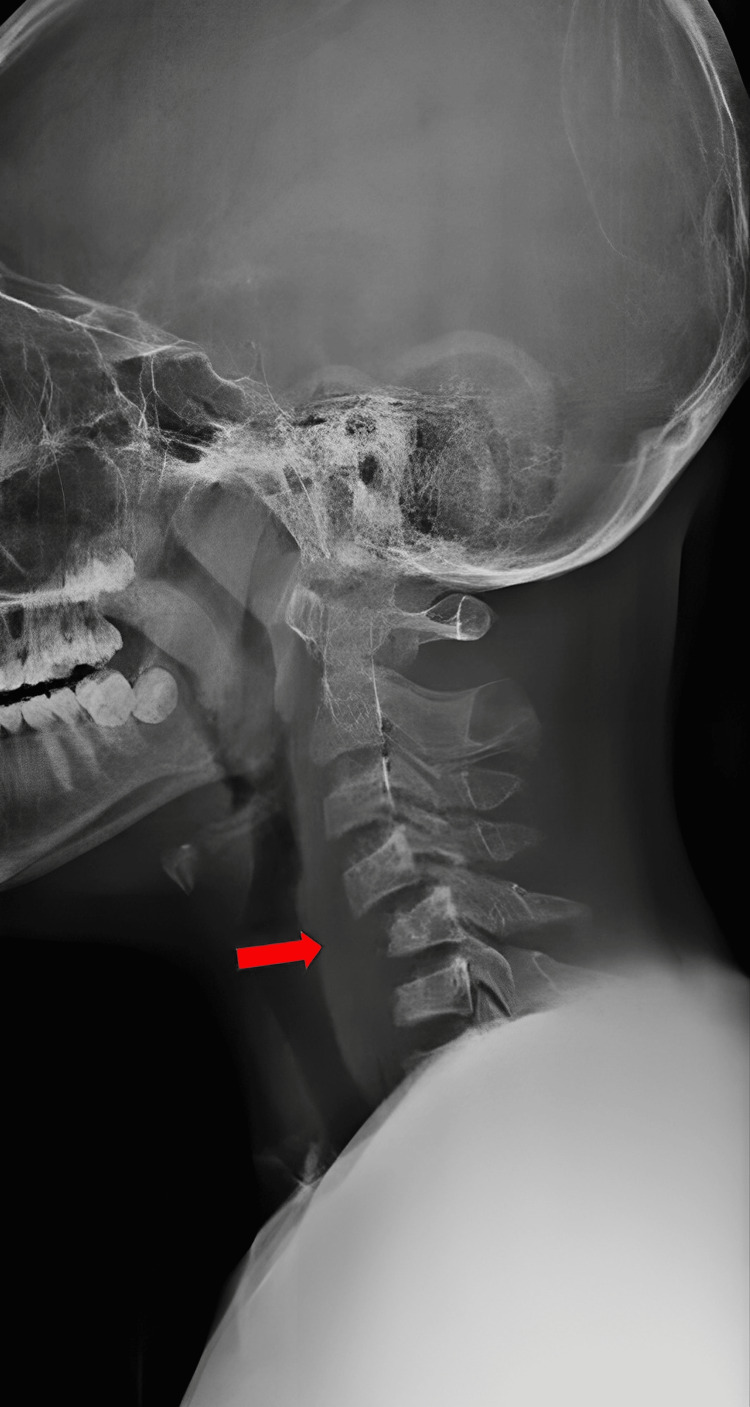
Lateral view of cervical X-ray with red arrow showing anterior translation of C4 over C5 with loss of cervical lordosis.

**Figure 2:**
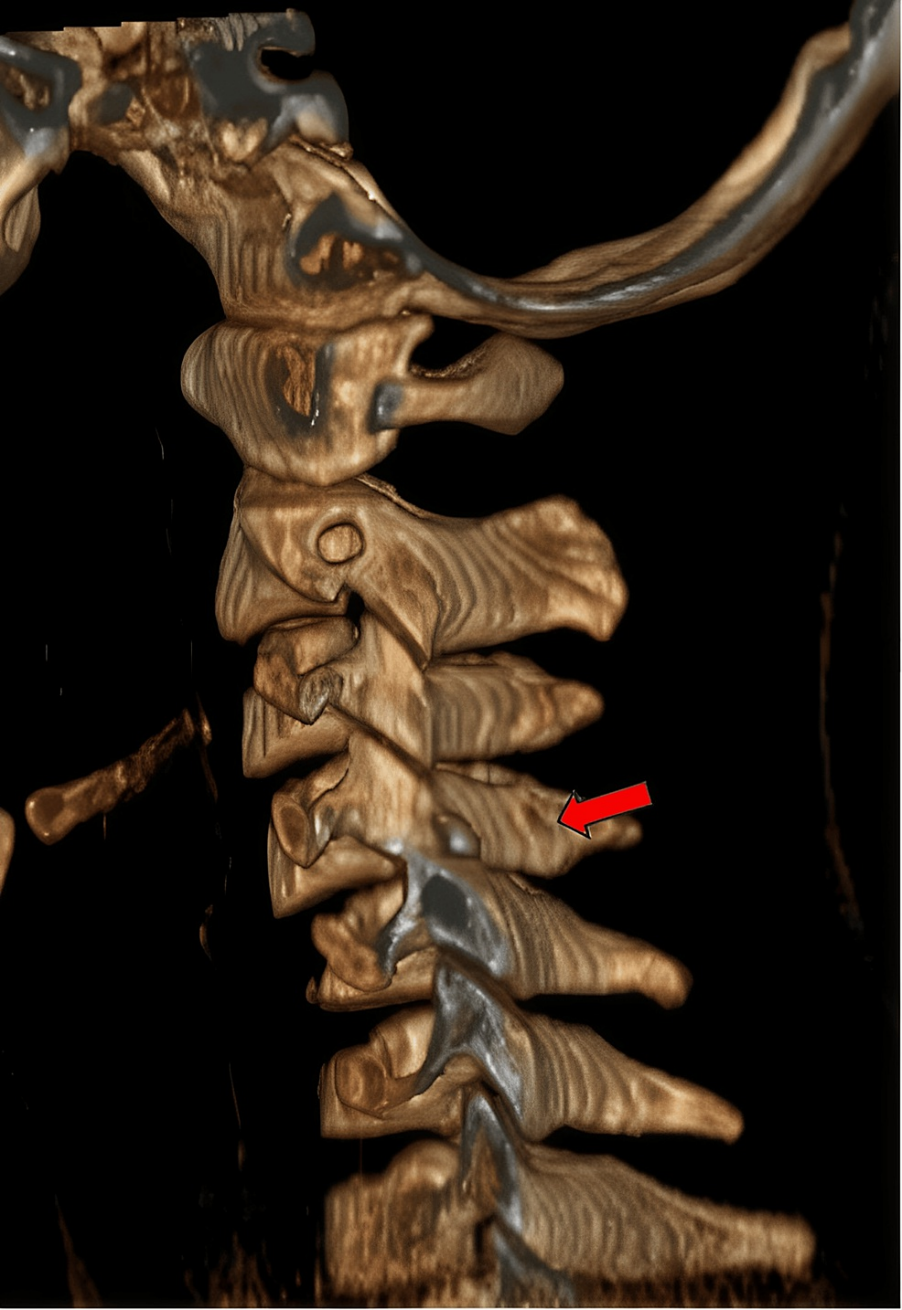
3D reconstruction image of CT scan with red arrow showing bilateral facet dislocation of C4/C5. 3D: three-dimensional; CT: computed tomography

**Figure 3:**
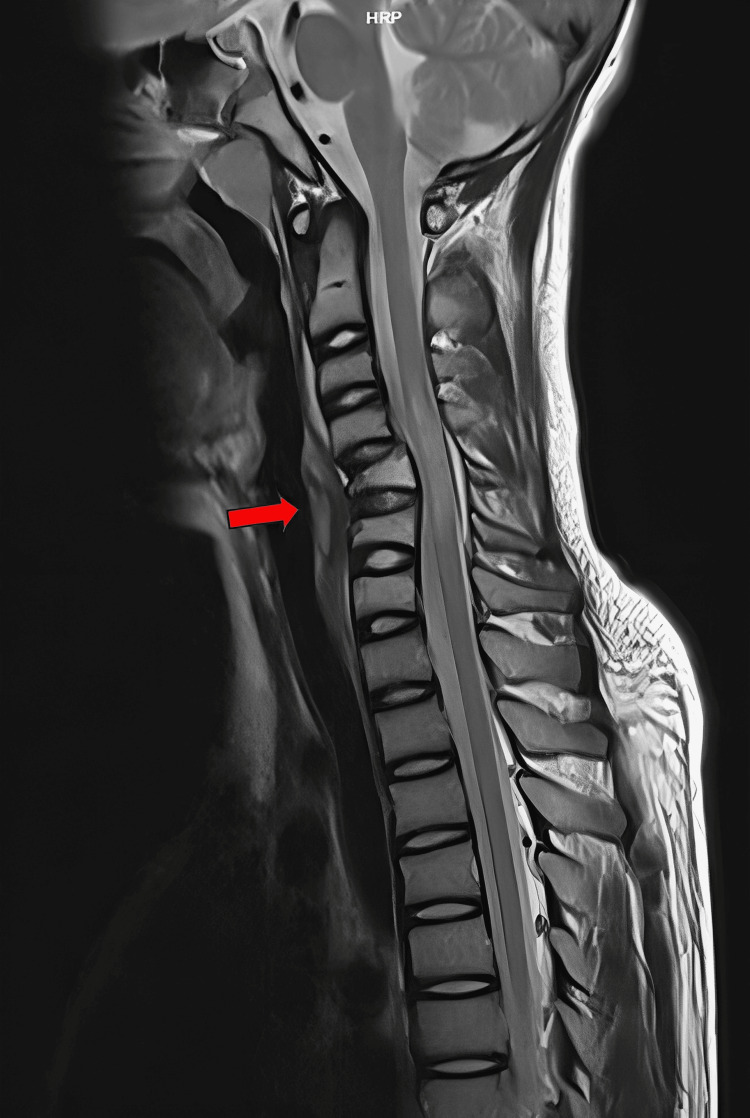
Sagittal view of cervical MRI with red arrow showing anterior dislocation of C4/C5 with bilateral perched facet joint and spinal cord oedema at the level of C4-C6 with intramedullary haemorrhage. MRI: magnetic resonance imaging

Plain lateral cervical radiographs (Figure 1) revealed anterior translation of C4 over C5 with 50% displacement, suggesting facet dislocation with loss of cervical lordosis. However, there was no significant increase in the anterior cervical soft tissue shadow. Further imaging with CT scans of the brain and cervical spine showed bilateral facet dislocation of C4/C5 (Figure 2) with no evidence of cervical spine fractures or brain injuries. Magnetic resonance imaging (MRI) revealed bilateral C4/C5 perched facet joints with spinal cord oedema from C4 to C6 and intramedullary haemorrhage but no spinal stenosis. There were no desiccated discs, posterior disc bulges, or bone marrow changes (Figure 3).

Treatment

**Figure 4:**
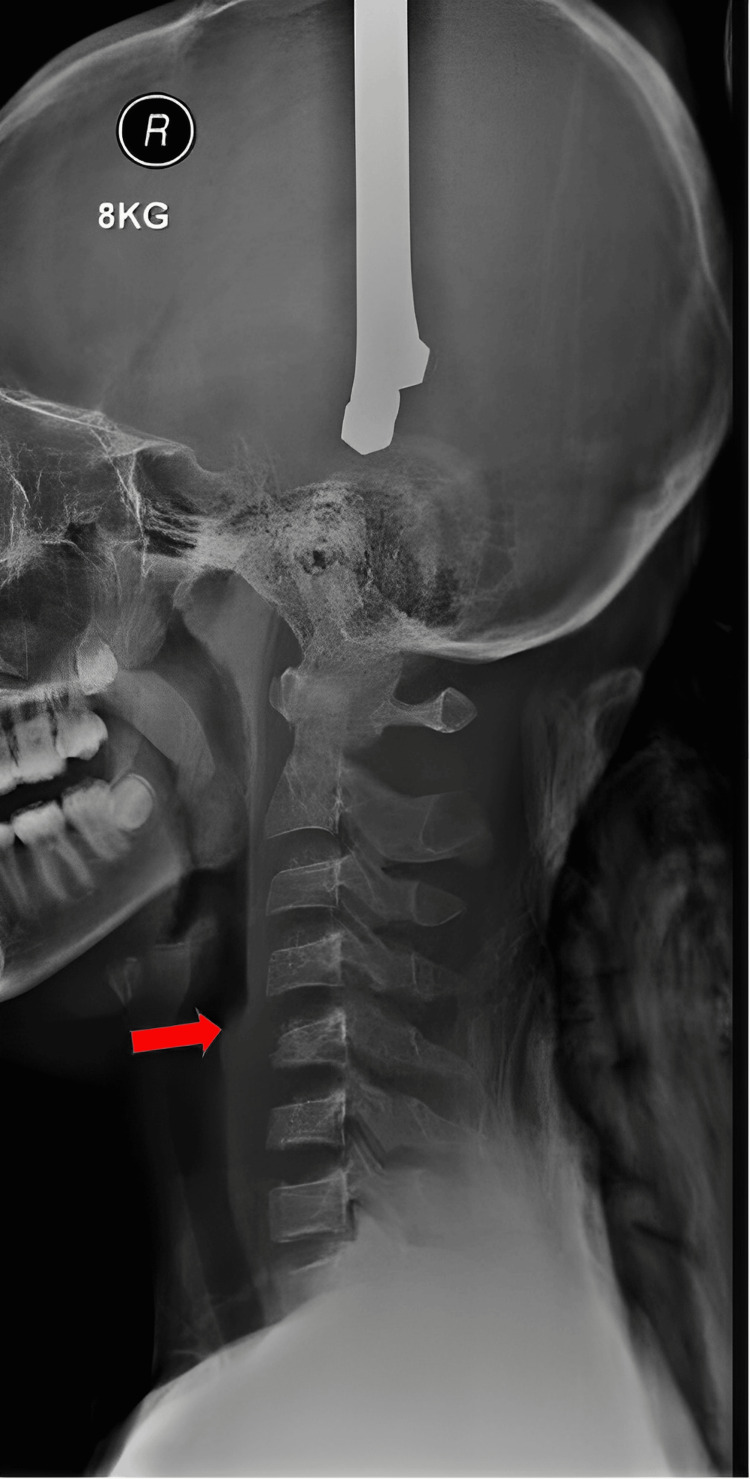
Lateral view of cervical X-ray on 8 kg of traction with red arrow showing reduction of C4/C5 facet dislocation.

**Figure 5:**
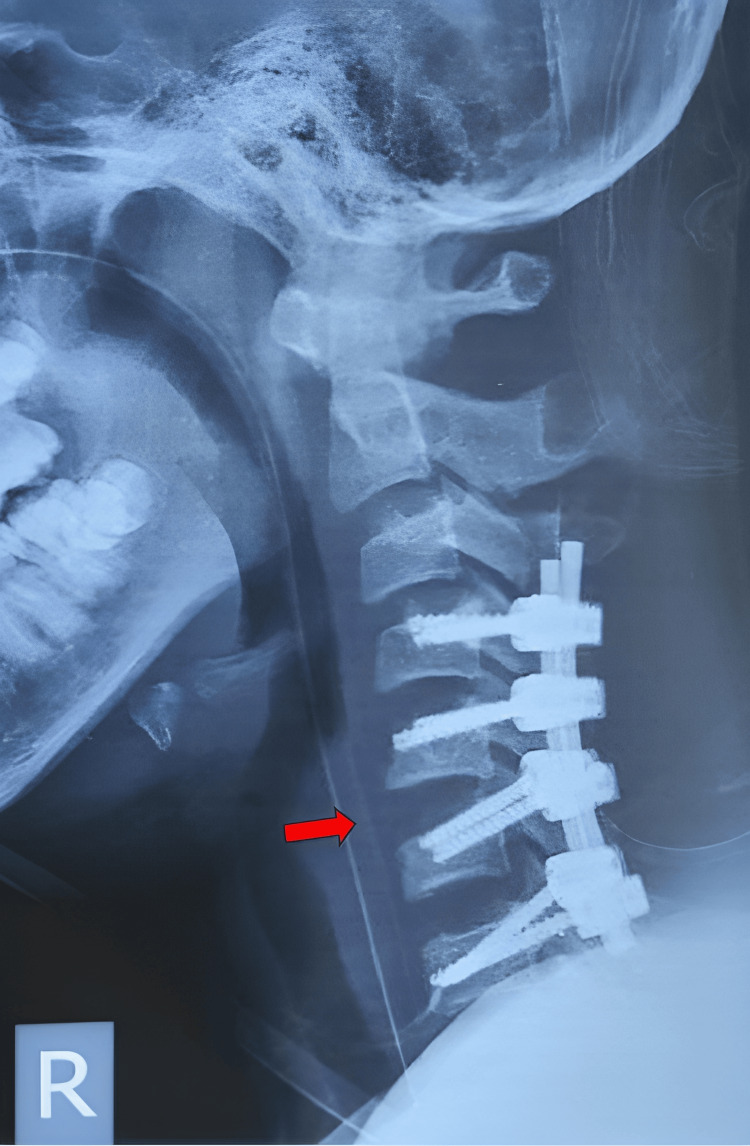
Lateral view of cervical X-ray postoperatively with red arrow showing restoration of cervical alignment with implant in situ.

The patient was immobilized with a cervical collar and started on intravenous (IV) dexamethasone 8 mg three times a day (TDS). He underwent both CT and MRI scans immediately after admission. Skull tong traction was applied for the gradual closed reduction of the dislocated bilateral facets of C4/C5. The initial weight of the skull tong was 2.5 kg, with an increment of 0.5 kg every 30 minutes, closely monitored for pain scores and neurological status, with repetitive lateral cervical X-rays. Closed reduction was successfully performed with a weight of 8 kg, resulting in the realignment of the cervical bodies of C4 and C5 (Figure 4). Due to the unstable cervical injury and incomplete neurological deficit, the patient underwent surgical intervention on the fourth day post trauma. He was treated with posterior spinal instrumentation and fusion of C4-C7, with laminectomy of C4-C6. Intraoperative findings revealed bilateral facet dislocation of C4/C5 with a torn capsule posteriorly. The spinal cord was bulging and pulsatile post laminectomy for decompression, with no intraoperative complications. A postoperative check X-ray showed acceptable reduction of the C4/C5 dislocation and stable fixation (Figure 5).

The patient showed favourable signs of neurological recovery, with improved muscle power in both upper limbs, from an overall MRC power grade of 2-3, one week after surgical intervention. Subsequently, he was transferred to the rehabilitation unit for aggressive therapy to optimize neurological recovery and prevent complications from being bedridden. At the six-week postoperative clinic follow-up, he reported minimal neck pain with no significant limitation of neck function. He could walk with a walking frame and had control over both micturition and defecation. Neurological examination revealed an improvement in central cord syndrome, progressing from ASIA Impairment Scale C to ASIA D neurological deficit with normal sensation. The cervical X-ray showed the implant in situ with good alignment.

At the six-month postoperative follow-up, the patient was doing very well, with no complaints. He was able to walk independently, with residual weakness in bilateral C8 and T1, rated at 4 on the MRC scale, compared to normal muscle power in other myotomes. The cervical X-ray revealed bone fusion over C4-C7.

## Discussion

Cervical spine trauma is a catastrophic injury resulting from shallow water diving, comprising approximately 2.5% of all cervical spinal trauma, as reported by Badman et al. [3]. Patients' average age across multiple series ranged from 27.7 [4] to 31.5 years, with 79.5% being adults aged 18-50 [5] and only 10-15% being adolescents aged 15-17 [5,6]. Notably, there are no reported diving injuries in young adolescents aged 12-14. Previous studies indicate that cervical facet subluxation without fracture is a rare occurrence in spinal cord injuries related to shallow water diving, constituting less than 10% compared to other common causes such as compression, burst, tear drop fracture, or fracture with subluxation [1,4,7].

Blanksby et al. reported that serious accidents during shallow water diving are influenced by factors such as reckless behaviour, misjudgement of water depth, and alcohol consumption [8]. Our case involves individuals in the early adolescent age group who lack awareness of the risks associated with shallow water diving, especially in recreational activities without proper supervision from family or caregivers, and who may not be familiar with the swimming area.

In addition to the catastrophic neurological complications of cervical injuries from diving, these incidents can lead to prehospital complications, such as drowning, resulting in aspiration and coma. Ull et al. reported that 40% of all cases require intubation and 16.7% require cardiopulmonary resuscitation [4]. Fortunately, our case did not involve complications from drowning. Instead, our focus was on managing unstable cervical spine injuries with neurological deficits to maximize the patient's recovery.

MRI serves as the most sensitive diagnostic tool for cervical spine injuries, especially in neurologically impaired patients. It plays a crucial role in evaluating disc herniation, ligamentous injuries, traumatic cord injuries, and the extent of cord oedema or hematoma before surgical intervention. Hart et al. suggested that timely MRI is essential to avoid unnecessary delays, which can hinder dislocation reduction and contribute to poorer outcomes [9].

Vaccaro et al. established the severity of cervical spine injuries can be assessed using the Subaxial Cervical Spine Injury Classification System (SLIC), aiming to incorporate injury pattern, severity, and neurological insult to define injury prognosis and guide treatment [10]. Patients with scores ≤ 3 are treated non-operatively, while patients with scores ≥ 5 are treated operatively. For patients with a score of 4, the treatment is guided by the surgeon's experience and preference, as well as the patient's comorbidities and other injuries. In our case, the SLIC score assessment was 9 due to facet dislocation and disrupted disco-ligamentous complex with incomplete neurological deficit, indicating the need for surgical stabilization with decompressive surgery even after successful closed reduction.

The optimal treatment strategy for cervical facet dislocation remains a matter of debate. Although the literature agrees on the role of closed reduction and surgical treatment for these injuries, there are still areas of debate regarding the superiority of one surgical approach over another as concluded by Mubark et al. [11]. Surgical approaches for unilateral or bilateral facet injuries include a stand-alone anterior approach, a stand-alone posterior approach, or a combined anterior and posterior approach. The choice of surgical approach depends on the degree of instability for unilateral or bilateral injuries, the degree of osteo-ligamentous injury, the presence of anterior disc herniation, and whether the dislocation is reducible from a single anterior or posterior approach alone [12].

AO Spine group concluded that no consensus exists regarding the preferred level of fixation for a posterior approach to subaxial facet dislocation [13]. In our case, a long-segment fixation involving four segments was performed due to a highly unstable injury with significantly disrupted osteo-ligamentous structures and bilateral facet dislocations based on the SLIC score. This was necessary to provide adequate stability post laminectomy of C4-C6 for the proper decompression of spinal cord oedema.

The treatment goals for cervical facet dislocation include reducing dislocation to restore normal cervical anatomy, providing cervical instrumentation for spinal stability, aiding in rehabilitation, and decompressing neural elements to enhance neurological recovery [14]. While there's no consensus on the timing of closed reduction, some authors recommend immediate reduction when it's safe. Performing closed reduction within four hours of injury is crucial for achieving a positive outcome, as delays beyond this time frame are associated with poorer results [15,16]. According to Furlan et al., early reduction and decompression within 24 hours have shown higher rates of neurological improvement compared to delayed intervention [17].

In our case, closed reduction successfully realigned the facet dislocation within eight hours post injury due to transportation delays from the district hospital. Surgical intervention occurred on the fourth day post trauma. Despite the delay in surgical stabilization and decompression, our patient achieved a good neurological outcome due to the early reduction of the dislocated segment and spinal cord oedema at the initial MRI, indicating the primary injury might be responsible for the incomplete neurological deficit with good recovery [18]. The patient's management involved counselling in accordance with CAse REport (CARE) guidelines.

While prompt management, accurate intervention, and aggressive rehabilitation can lead to favourable neurological and functional outcomes, the ultimate priority lies in reinforcing effective preventive strategies to reduce the incidence and impact of shallow water diving accidents. Preventing diving accidents requires an emphasis on education and raising awareness about the risks associated with negligent and reckless behaviour in diving activities [19]. Strategies could involve the compulsory installation of warning signs, the presence of lifeguards, educational programs in schools, regular media campaigns, and the requirement for adult supervision for young adolescents.

## Conclusions

Dangerous diving can result in severe spinal injuries with long-lasting consequences, including permanent physical disabilities that necessitate lifelong rehabilitation and care. The accurate diagnosis, prompt management, accurate intervention, and aggressive rehabilitation can lead to favourable neurologic and functional outcome of cervical spine injury. Preventing these incidents and minimizing the complications of such injuries depend on primary prevention efforts, focusing on increasing awareness of the dangers associated with diving accidents.
